# Pharmacological Basis for Traditional Use of the *Lippia thymoides*

**DOI:** 10.1155/2015/463248

**Published:** 2015-03-29

**Authors:** Fabrício Souza Silva, Pedro Modesto Nascimento Menezes, Pedro Guilherme Souza de Sá, André Luís de Santana Oliveira, Eric Alencar Araújo Souza, Vinicius Martins Bamberg, Henrique Ribeiro de Oliveira, Sheilla Andrade de Oliveira, Roni Evêncio e Araújo, Ana Paula Trovatti Uetanabaro, Tânia Regina dos Santos Silva, Jackson Roberto Guedes da Silva Almeida, Angélica Maria Lucchese

**Affiliations:** ^1^Núcleo de Estudos e Pesquisas em Plantas Medicinais, Colegiado de Ciências Farmacêuticas, Universidade Federal do Vale do São Francisco, Avenida José de Sá Maniçoba, s/n, Centro, 56304-917 Petrolina, PE, Brazil; ^2^Programa de Pós-Graduação em Biotecnologia, Departamento de Ciências Biológicas, Universidade Estadual de Feira de Santana, Avenida Transnordestina, s/n, Novo Horizonte, 44036-900 Feira de Santana, BA, Brazil; ^3^Laboratório de Química de Produtos Naturais e Bioativos, Departamento de Ciências Exatas, Universidade Estadual de Feira de Santana, Avenida Transnordestina, s/n, Novo Horizonte, 44036-900 Feira de Santana, BA, Brazil; ^4^Laboratório de Imunopatologia e Biologia Molecular, Departamento de Centro de Pesquisa Aggeu Magalhães, Fundação Oswaldo Cruz, Avenida Professor Moraes Rego, s/n, Campus da UFPE, Cidade Universitária, 50670-420 Recife, PE, Brazil; ^5^Laboratório de Microbiologia da Agroindústria, Universidade Estadual de Santa Cruz, Ilhéus, BA, Brazil; ^6^Departamento de Ciências Biológicas, Universidade Estadual de Feira de Santana, Feira de Santana, BA, Brazil; ^7^Programa de Pós-Graduação em Recursos Naturais do Semi-Árido, Universidade Federal do Vale do São Francisco, Avenida José de Sá Maniçoba s/n, Centro, 56304-917 Petrolina, PE, Brazil

## Abstract

The aim of this study was to evaluate crude extracts and fractions from leaves and stems of* Lippia thymoides* and to validate their use in folk medicine.* In vitro* antioxidant and antimicrobial activities and* in vivo* wound healing in rats, baker yeast-induced fever in young rats, and acute oral toxicity in mice assays were realized. The crude extracts and their dichloromethane and ethyl acetate fractions had potent radical-scavenging activity against the DPPH but were not effective in the *β*-carotene bleaching method. The dichloromethane fraction from the leaves extract showed the broadest spectrum of activity against* S. aureus*,* B. cereus*, and* C. parapsilosis*. The animals treated with crude extracts showed no difference in wound healing when compared with the negative control group. The crude extract from leaves (1200 mg/kg) has equal efficacy in reducing temperature in rats with hyperpyrexia compared to dipyrone (240 mg/kg) and is better than paracetamol (150 mg/kg). In acute toxicity test, crude extract of leaves from* Lippia thymoides* exhibited no mortality and behavioral changes and no adverse effects in male and female mice. This work validates the popular use of* Lippia thymoides* for treating the wound and fever, providing a source for biologically active substances.

## 1. Introduction

The genus* Lippia* has a great importance for Brazilian flora due to its economic utilization as a condiment and its use in traditional medicine [1]. This genus includes about 200 taxa, including herbs, shrubs, and small trees, distributed in the tropics and subtropics from America and Africa, being common in the Brazilian savannah and rocky fields [2].

Use of plants belonging to the genus* Lippia* in folk medicine follows a general profile, mainly utilizing leaves or flowers and aerial parts prepared in the form of infusion or decoction and administered orally. The main indications are for the treatment of diseases of the respiratory and digestive systems and for the treatment of infections [3]. Although popular use of* Lippia* species is registered in various studies [4–7], there are few correlated pharmacological data and folk medicine. Furthermore, most studies are concentrated in some species, for instance,* L. sidoides*,* L. alba*,* L. dulcis*, and* L. graveolens*.

*Lippia thymoides* Mart. & Schauer is a shrub of two meters in length, very thin, erect, branched, with small and aromatic leaves, white or lilac flowers, and occurring in the Caatinga vegetation from states of Bahia and Minas Gerais in Brazil. It is popularly known as “alecrim-do-mato” or “alecrim-do-campo,” being utilized in religious rituals and folk medicine to treat wounds, fever, bronchitis, rheumatism, headache, and weakness [8, 9]. Recently, Pinto et al. [10] reported that crude extracts from* L. thymoides* showed antimicrobial activity, but this study was restricted only in agar disk diffusion method. Thus, considering the popular uses and absence of pharmacological reports of this species in the literature, the aim of this study was to evaluate crude extracts and fractions from* L. thymoides* by* in vitro* and* in vivo *assays befitting popular indication and to validate their use in traditional medicine.

## 2. Material and Methods

### 2.1. Drugs and Reagents

The following reference chemicals were used in the experiments: butylated hydroxytoluene (BHT), butylated hydroxyanisole (BHA), resazurin, 2,3,5-triphenyltetrazolium chloride (TTC), chloramphenicol, nystatin, quercetin, pyrogallol Cremophor EL, all provided by Sigma-Aldrich (St. Louis, MO, USA); ascorbic acid (AAc), purchased from Dinâmica (Diadema, São Paulo, Brazil); linoleic acid, obtained from Vetec (Duque de Caxias, Rio de Janeiro, Brazil); 2,2-diphenyl-1-picrylhydrazyl (DPPH), provided by FLUKA (St. Louis, MO, USA); silver sulphadiazine 2% (União Química, Brasília, Distrito Federal, Brasil); paracetamol (Johnson & Johnson, São José dos Campos, São Paulo, Brazil); dipyrone (Sanofi Aventis, São Paulo, Brazil); ketamin (Cristália, Itapira, São Paulo, Brazil); and xylazine (Intervet Schering-Plough, São Paulo, Brazil). Stock solutions of these chemicals were prepared with suitable solvent and dilutions were made fresh on the day of experiment. Mueller-Hinton Agar and Mueller-Hinton Broth culture mediums were both purchased of HiMedia (Mumbai, India) and prepared with sterile distilled water.

### 2.2. Plant Material

*L. thymoides* was collected in the Feira de Santana, Bahia, Brazil (12°11′45′′ S latitude and 38°58′05′′ W longitude). The voucher specimen was deposited at the Herbarium of Universidade Estadual de Feira de Santana (HUEFS) under number 77554. The sample was identified by Dr. Tânia Regina dos Santos Silva. Leaves were separated from the stems and air-dried at room temperature, protected from light. Methanol extracts were prepared with powdered stems and leaves by exhaustive maceration in ultrasonic bath, at 37°C. At each interval of 30 minutes, the solvent was filtered, and this step was repeated at least five times. The solvent was removed by evaporation under vacuum on reduced pressure at 40–45°C using a rotary evaporator. Residual methanol was evaporated at room temperature, and water was lyophilized to obtain crude extracts of leaves (TF) and stems (TC), with yields of 35% and 4%, respectively. Then, TF and TC were redissolved in methanol and partitioned with solvents hexane, dichloromethane and ethyl acetate, to obtain respective fractions, as shown in Table 1.

### 2.3. Animals

Adults (6 to 10 weeks and 250–300 g) and young (27 to 30 days and 60–90 g) male Wistar rats and Swiss mice of both sexes (4 to 8 weeks and 30–40 g) used in the assays were bred and housed at the animal house of Universidade Federal do Vale do São Francisco. All experiments performed complied with the rulings of the US guidelines (NIH publication no. 85-23, revised in 1985) and approved by the Ethics Committee on Animal Use of the Universidade Estadual de Feira de Santana (protocol no. 018/2009). Animals were kept under conditions of controlled temperature (23–25°C), 12-hour light/dark cycles with food and water* ad libitum*.

### 2.4. Total Phenolic Content

The amount of total phenolics of crude extracts and fractions (TF, TFH, TFD, TFA, TC, TCH, TCD, and TCA) was determined with the Folin-Ciocalteu reagent using the method of Slinkard and Singleton [11]. An aliquot (40 *μ*L) of a suitable diluted extracts was added to 3.16 mL of distilled water and 200 *μ*L of the Folin-Ciocalteu reagent and mixed well. The mixture was shaken and allowed to stand for 6 min, before adding 600 *μ*L of sodium carbonate solution, and shaken to mix. The solutions were left at 20°C for 2 hours and the absorbance of each solution was determined at 765 nm against the blank and plot absorbance versus concentration. Total phenolic contents of the crude extracts and fractions were expressed as mg gallic acid equivalents per gram (mg GAE/g) through the calibration curve with gallic acid. The calibration curve range was 50–1000 mg/L (*R* ^2^ = 0.9991). All samples were performed in triplicate in three different experiments.

### 2.5. DPPH Radical Scavenging Activity

The antioxidant activity by DPPH free radical scavenging was performed as described by Brand-Williams et al. [12]. Crude extracts, fractions, and positive controls were dissolved in EtOH, with sample stock solutions (1.0 mg/mL) of the crude extracts and fractions (TF, TFH, TFD, TFA, TC, TCH, TCD, and TCA) and positive controls (AAc, BHA, and BHT) diluted to final concentrations of 243, 81, 27, 9, 3, and 1 *µ*g/mL. One mL of a 50 *µ*g/mL DPPH ethanol solution was added to 2.5 mL of sample solutions previously prepared and allowed to react at room temperature. After 30 min the absorbance values were measured at 518 nm and converted into the percentage antioxidant activity. The crude extracts and fractions plus diluents were used as blanks and DPPH solution without sample solutions was used as negative control. The antioxidant activity of DPPH assay was calculated using the formula % of activity = [(absorbance of the control − absorbance of the sample)/absorbance of the control] × 100, and concentration that caused 50% of the scavenging (EC_50_) was calculated by nonlinear curve fitting. Assay was performed in triplicate in three different experiments.

### 2.6. *β*-Carotene Bleaching Test

This assay was performed as described by Miguel [13]. *β*-Carotene (2 mg) was dissolved in 10 mL chloroform and to 2 mL of this solution, linoleic acid (40 mg) and Tween 40 (400 mg) were added. Chloroform was evaporated under vacuum at 40°C and 100 mL of distilled water was added, and then the emulsion was vigorously shaken during two minutes. The emulsion (3.0 mL) was added to a tube containing 0.12 mL of solutions 1 mg/mL of positive controls (AAc, BHA, and BHT) and crude extracts and fractions (TF, TFH, TFD, TFA, TC, TCH, TCD, and TCA). The absorbance was immediately measured at 470 nm and the test emulsion was incubated at a water bath at 50°C for two hours, when the absorbance was measured again. In the negative control, the essential oils were substituted with an equal volume of diluent. The percentage antioxidant activity was evaluated in terms of the bleaching of the *β*-carotene. The antioxidant activity of bleaching assay was calculated using the following formula: % activity = [1 − (*A*_0_ − *A*_*t*_)/(*A*_0_^0^ − *A*_*t*_^0^)] × 100, where *A*_0_ is the initial absorbance, *A*_*t*_ is the final absorbance measured for the test sample, *A*_0_^0^ is the initial absorbance, and *A*_*t*_^0^ is the final absorbance measured for the negative control (blank). Assay was performed in triplicate in three different experiments.

### 2.7. Antimicrobial Assays

The antimicrobial assays were performed by minimum inhibitory concentration (MIC) and minimum microbicidal concentration (MMC) methods, according to what is approved by Clinical and Laboratory Standards Institute [14, 15]. Crude extracts, fractions, and positive controls were dissolved in dimethylsulfoxide (DMSO) 50% v/v in distilled water. The microorganisms tested* Escherichia coli *(CCMB 261) resistant to sulfonamide and sensible to trimethoprim,* Staphylococcus aureus *(CCMB 262) resistant to streptomycin and dihydrostreptomycin,* Micrococcus luteus* (CCMB 283),* Salmonella choleraesuis* (CCMB 281),* Bacillus cereus* (CCMB 282),* Candida albicans *(CCMB 286) resistant to fluconazole e amphotericin B, and* Candida parapsilosis *(CCMB288) were obtained from Culture Collection of Microorganisms of Bahia (CCMB). In 96-well plates, 90 *μ*L of the extracts and fractions samples (TF, TFH, TFD, TFA, TC, TCH, TCD, and TCA) and 90 *μ*L of Müeller-Hinton broth (2x concentrated) were conditioned in the first well and the serial dilutions were carried out in all subsequent wells. The range of evaluated extract concentration was 22.20 to 0.005 mg/mL. Cultures of 18 hours (bacteria) and 36 hours (yeast) were collected in a saline solution 0.45% and 10 *μ*L of microorganism suspension at 1.5 × 10^6^ CFU/mL (bacteria) and 5 × 10^5^ CFU/mL (yeast) was added in each well. The MIC of the DMSO/water solution and positives controls chloramphenicol (20 mg/mL) and nystatin (10 mg/mL) was also determinate. Controls of the microbial strains viability, sample sterility, and water were also performed. After incubation (24 hours at 37°C for bacteria and 48 hours at 28°C for yeast), 30 *µ*L of resazurin (0.01% w/v) was added in each well. The MIC was defined as the lowest concentrations which did not show any growth of tested organism.

To confirm the activity, all wells of MIC test which did not show any growth of the bacteria fungus after the incubation period were subcultured onto the surface of the freshly prepared Mueller-Hinton Agar plates and incubated at 37°C for 24 hours (bacteria) or 48 hours at 28°C (fungus). The MBC was recorded as the lowest concentration of the sample that did not permit any visible bacteria colony growth on appropriate agar plate after the period of incubation.

### 2.8. Wound Healing Assay in Rats

This assay was performed according to Panchatcharam et al. [16] with small modifications. Adults Wistar rats were divided into 2 groups of 25 animals for evaluation during 14 and 21 days. Each group was separated in subgroups of 5 animals maintained individually: NC, negative control treated with vehicle; TF100, treated with TF 100 mg/mL; TF200, treated with TF 200 mg/mL; TC100, treated with TC 100 mg/mL; TC200, treated with TC 200 mg/mL. Crude extracts were dissolved in DMSO 10% v/v in EtOH. After anesthesia by ketamine 75 mg/kg and xylazine 10 mg/kg, the backs of animals were shaved and sterilized with 70% EtOH. Using 20 mm punch biopsies, a full-thickness excisional wound (round wound) was realized and treated topically during 14 or 21 days with the application 100 *µ*L/wound of each extract tincture or silver sulphadiazine 2% (positive control). On the 14th or 21st day, the animals were anesthetized with thiopental 25 mg/kg and euthanized by cervical dislocation. Tissues from the wound site of each animal were removed for histopathological analysis and for total collagen determination.

Skin specimens from treated and untreated rats were collected in 10% buffered formalin and after the usual processing, 5 *µ*m-thick sections were cut and stained with Masson Trichrome. Quantitative analysis was performed by morphometry using a semiautomatic Image Processing and Analysis System Leica Qwin plus, version 2.6 (Leica, Cambridge, England). Areas of fibrous tissue both in treated groups as control groups were detected and measured to obtain the percentage of area tissue detected in relation to microscopically field examined.

### 2.9. Baker Yeast-Induced Fever Assay in Rats

Young male Wistar rats were used in this assay, according to method established by Tomazetti et al. [17]. Animals were housed in 9 groups of 5, which were treated orally: negative control treated with distilled water; positive control treated with paracetamol 150 mg/kg; positive control treated with dipyrone 240 mg/kg; treated with TF 300 mg/kg; treated with TF 600 mg/kg; treated with TF 1200 mg/kg; treated with TC 300 mg/kg; treated with TC 600 mg/kg; treated with TC 1200 mg/kg. Crude extracts and positive controls were dissolved in distilled water at moment of administration. To avoid temperature variation by stress, animals were habituated to the injection intraperitoneally with sterile 0.9% NaCl for 2 days before experiments were carried out. Rectal temperature (*T*_*R*_) was measured by inserting a lubricated digital thermometer (0.1°C precision) 2.8 cm into the rectum of the animal. Immediately after measuring the initial basal rectal temperature at day of experiment, the animals were injected with baker yeast (135 mg/kg, i.p.). The animals had their *T*_*R*_ measured for 4 h, and after the fourth *T*_*R*_ measurement they were administered with water, paracetamol, dipyrone, TC, or TF. *T*_*R*_ was recorded every hour up to 8 hours after the drug injections. *T*_*R*_ variation (Δ*T*_*R*_) to each animal was calculated using the following formula: Δ*T*_*R*_ = *T*_*Rt*_ − *T*_*R*0h_, where *T*_*Rt*_ is *T*_*R*_ measured in each hour (*t* = 1–12 h) and *T*_*R*0h_ is *T*_*R*_ measured before injection of baker yeast.

### 2.10. Acute Toxicity Testing

Male and female Swiss mice were divided into different groups of 6 animals each. The test was performed using doses of crude extract from leaves (TF) at 5, 1.7, or 0.6 g/kg given orally. Crude extract was dissolved in distilled water at the moment of administration, and the maximum volume utilized was 200 *µ*L. Another group of mice was administered saline at the same volume as the negative control. The animals were allowed food and water* ad libitum* and kept under regular observation for 0.5, 1, 2, 4, 12, and 24 hours to observe their piloerection, convulsions, hypnosis, respiratory frequency, changes in exploratory behavior, aggressiveness, and blindness, while lethality was monitored up to 14 days.

### 2.11. Statistical Analysis

All the numeric data were expressed as mean ± standard error of the mean (SEM). The statistical parameter applied was the student *t*-test and analysis of variance (ANOVA) following Tukey posttest, being considered as significantly different *P* < 0.05.

## 3. Results

### 3.1. Total Phenolics Content

The content of total phenolics varied according to part of the plant and polarity of solvent used and ranged from 14.5 to 213.6 mg GAE/g (Table 2). Total phenolic content was significantly higher in TF than TC and low levels were found in hexane fractions TFH and TCH. Among fractions from crude extracts, TFA and TCA had considerably high contents of phenolic substances when compared with crude extracts TF and TC, respectively.

### 3.2. Antioxidant Activity

Antioxidant activities of crude extracts and fractions from leaves and stems of* L. thymoides* are shown in Table 2. In DPPH scavenging method there was no significant statistical difference between TF (EC_50_ = 15.4 ± 1.6 *μ*g/mL) and TC (EC_50_ = 18.4 ± 2.4 *μ*g/mL) activity. The ethyl acetate fractions from leaves and stems extracts showed the greatest potency (TFA EC_50_ = 5.7 ± 0.4 *μ*g/mL and TCA EC_50_ = 4.5 ± 0.2 *μ*g/mL) among the extracts and fractions and exhibited the greatest radical-scavenging activity. When compared to the activity of natural and synthetic standards, such as ascorbic acid (EC_50_ = 5.1 ± 1.1 *μ*g/mL), BHT (EC_50_ = 13.3 ± 1.0 *μ*g/mL) and BHA (EC_50_ = 4.0 ± 0.6 *μ*g/mL), DPPH scavenging activity of methanolic crude leaves and stems extracts, and respective ethyl acetate and dichloromethane fractions, there were no significant statistical difference. On the other hand,* L. thymoides* crude extracts and fractions were not effective in *β*-carotene bleaching method, with maximum inhibition percentage at about 38%.

### 3.3. Antimicrobial Activity

Antimicrobial activity of crude extracts and fractions from leaves and stems of* L. thymoides* are shown in Table 3. Among all samples tested and microorganisms used, the TFD fraction showed the broadest spectrum of activity, being microbicidal against* S. aureus *(MMC of 0.69 mg/mL against CCMB 262 and 1.39 mg/mL against CCMB 263),* B. cereus* (MMC of 0.17 mg/mL), and* C. parapsilosis *(MMC of 1.28 mg/mL), whereas, against* M. luteus* (MIC of 0.69 mg/mL) and* C. albicans* (MIC of 2.48 mg/mL), it only inhibited growth. Other fractions from leaves and stems, as TFA, TCD, and TCA, presented antimicrobial activity against* S. aureus*,* B. cereus*, and* M. luteus*. Crude extracts TF and TC were less potent than the respective fractions. In general,* L. thymoides* showed potent antimicrobial activity against Gram-positive bacteria and weak activity against Gram-negative bacteria and was inefficacious against fungi.

### 3.4. Wound Healing Activity

**Figure 1:**
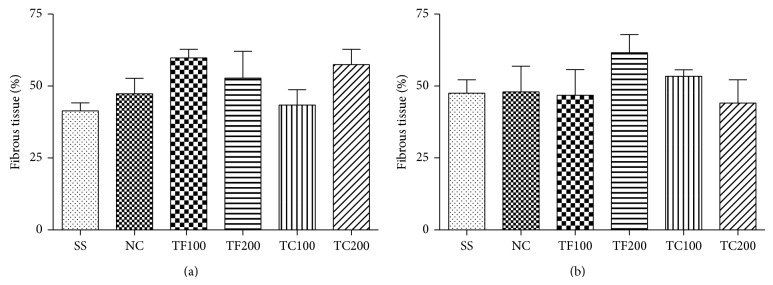
Effect of crude extracts of the leaves and stems from* L. thymoides* on the fibrous tissue deposition in rats. In (a) animals treated for 14 days and (b) animals treated for 21 days. Data are presented as mean ± SEM (*n* = 5); SS, silver sulphadiazine 2%; NC, negative control; TF100, animals topically treated with crude extract from leaves (TF) at 100 mg/mL; TF200, animals topically treated with TF at 200 mg/mL; TC100, animals topically treated with crude extract from leaves (TC) at 100 mg/mL; TC200, animals topically treated with TC at 200 mg/mL.

In the excision wound model in rats, the animals treated with crude extracts showed no difference in the epithelization period and deposition of collagen when compared with negative control group, as shown in Figure 1. Histopathological analysis of skin specimens preparations showed not difference between animals treated with* L. thymoides* extracts and negative control that received only a water-alcohol solution.

### 3.5. Antipyretic Activity

**Figure 2:**
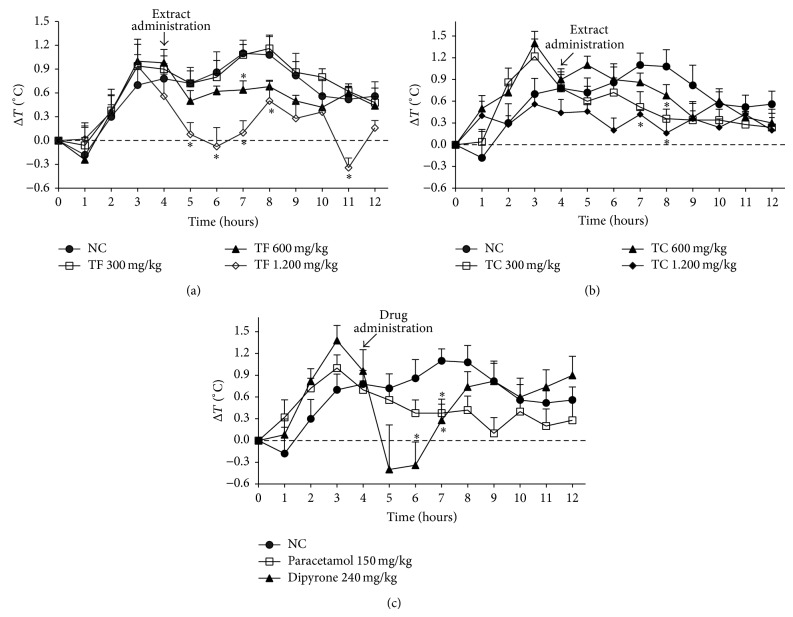
Effect of crude extracts of the leaves and stems from* L. thymoides* on hyperpyrexia-induced fever in young rats. (a), (b), and (c) represent groups of the animals treated with TF, TC, and positive controls (paracetamol and dipyrone), respectively. Data are presented as mean ± SEM (*n* = 5); ∗ represents temperature variation that was statistically different (*t* test, *P* < 0.05) when compared with the negative control (NC). TF, crude extract from leaves; TC, crude extract from stems.

Figure 2 shows antipyretic effect of crude extracts from* L. thymoides* assessed using baker yeast-induced fever in rats. Administration of TF 1200 mg/kg reduced significantly (*P* < 0.05) temperature at 5 to 8 h when compared to negative control (NC) group that received only distilled water and TC 1200 mg/kg significantly (*P* < 0.05) reduced temperature at 7 to 8 hours compared to negative control group. At dose 600 mg/kg, TF and TC reduced temperature only at 7 and 8 hours, respectively, when compared to NC group. At a dose of 300 mg/kg both TF and TC did not show significant reduction in temperature at any time. The TF 1200 mg/kg has equal efficacy in reducing temperature in rats with hyperpyrexia, compared with dipyrone 240 mg/kg at 5 to 8 hours and better than paracetamol 150 mg/kg.

### 3.6. Acute Toxicity Analysis of TF

Over the study duration of 14 days, there were no deaths recorded in the male and female animals given 5, 1.7, or 0.6 g/kg of the crude extract from leaves of the* L. thymoides* orally. During the observation period animals did not produce any variations in the general appearance.

## 4. Discussion

The aim of this study was to validate the popular use of the* L. thymoides* for treatment of wounds and fever, with the utilization of* in vitro* and* in vivo* experimental models that correlate with these indications. Initially, extracts and fractions from the leaves and stems of this species were characterized qualitatively, with the determination of total phenolic content, since studies that identified fixed constituents of* Lippia* species showed that there is a pattern in the presence of phenolic acids, flavonoids, and phenylpropanoids [18–21]. In* L. thymoides*, it was found that total phenolic content was significantly higher in extracts from leaves than the stems, with concentration of phenolic constituents in polar organic fractions such as TFA and TCA. The values of total phenolic content were similar or superior to other sources of phenolic compounds, such as green tea, black tea, cabbage, carrots, broccoli, apple, cranberry, and grape [22].

As the presence of phenolic compounds is related to antioxidant activity from plant metabolites and the formation of reactive oxygen species (ROS) is involved in various pathological events such as wound healing and fever [23, 24], the antioxidant potential of extracts and fractions of* L. thymoides* was evaluated. Thus, the chemical mechanisms involved in the scavenging of radical DPPH^•^ indicate that antioxidant activity of plant extracts may be related to the presence of substances with hydroxyl groups, such as phenolic compounds [25]. The reaction of DPPH^•^ with phenols (ArOH) occurs through the donation of the proton H or electron transfer from ArOH or its phenoxide anion (ArO-) to DPPH^•^ [26], which can be observed on the antioxidant potency and efficacy of polar fractions such as TFA and TCA, which were the fractions with the highest phenolic content.

Another method used to evaluate the antioxidant potential of* L. thymoides* was the ability of the extracts or fractions of this plant to sequester or neutralize peroxide radicals formed from linoleic acid in emulsion. These radicals attack the double bonds of *β*-carotene and thus induce a loss of orange color, but the presence of an antioxidant can prevent this attack and keep the carotenoid coloration [13]. As this assay involves formation of an oil-in-water emulsion, the ability of a substance to prevent the oxidation of *β*-carotene should be more effective among the less polar antioxidants, according to the theory of polar paradox [27]. However, several studies have shown that the physicochemical characteristics of antioxidants, which determine the inhibition or stabilization of lipid oxidation, do not depend only on polarity, but also on various factors such as concentration of the constituent, temperature, and characteristics of the test system, among others [28–30]. The extracts and fractions of the* L. thymoides* were not effective in preventing the oxidation of *β*-carotene. Correlating the results of total phenolic content, DPPH^•^ radical scavenging, and inhibition of oxidation of *β*-carotene, data show that TCA and TFA fractions may contain phenolic constituents which are capable of stabilizing radicals already formed but are not effective in preventing oxidation caused by lipid peroxides.

One of the indications of the species* L. thymoides* in the folk medicine is to treat wounds; however, it is not known whether this use is related to the antiseptic effect or stimulating healing, for example, increasing collagen deposition. Thus,* in vitro* antimicrobial activity of extracts and fractions from the leaves and stems against bacteria and fungi showed that TF had no significant effect and TC only inhibited growth of* S. aureus* CCMB 262. On the other hand, TFD, TFA, TCD, and TCA fractions were potent against strains of* S. aureus* (CCMB 262 and CCMB 263) and* Bacillus cereus* CCMB 282. Only TFD showed considerable fungicidal activity against* Candida parapsilosis* CCMB288. It is interesting to observe that the fractions with higher total phenolic content showed significant antimicrobial activity, indicating a correlation between the presence of these classes of secondary metabolites with the effect on microorganisms. Phenolic compounds have a broad spectrum of action against bacteria and fungi, acting from the plasma membrane of microorganisms to inactivation of essential enzymes in metabolic processes [31].

The data presented in this work identified the species* L. thymoides* as a possible source of compounds with antimicrobial activity mainly against Gram-positive bacteria such as* S. aureus*, an opportunistic pathogen that colonizes asymptomatically the skin and mucous membranes of healthy human and is the main cause of infections of skin wounds [32]. Thus, the* in vitro* antimicrobial activity of* L. thymoides* is consistent with the popular use of this species in the treatment of skin infections caused primarily by bacteria. The selectivity of action of the fractions from extracts on Gram-positive bacteria indicates that the morphology of microorganisms can drastically influence the antimicrobial activity and, probably, the presence of outer membrane exclusively in Gram-negative bacteria influenced the action of extracts of* L. thymoides*.

Following the aim of correlating the popular use of* L. thymoides* with experimental data, the healing of skin wounds in rats was evaluated with application topically of TF and TC extracts. It was observed that, at the concentrations tested, there was no significant difference between animals treated with crude extracts and those who received only the vehicle. The healing of acute wounds such as traumatic injuries and burns or surgically created wounds involves three phases: (1) inflammatory phase involves the vascular response characterized by blood coagulation and hemostasis, including cellular responses such as leukocyte infiltration and release cytokines; (2) proliferative phase involves the formation of the epithelium to cover the wound and the granulation tissue with proliferation of fibroblasts, extracellular matrix deposition, and development of new blood vessels; (3) remodeling phase involves restoring the structural and functional integrity of the tissue [33, 34]. The interest in evaluating the influence of extracts of* L. thymoides* in accelerating the healing process led to the decision to investigate the effect of TF and TC on the proliferative phase by determining collagen deposition after 14 and 21 days of treatment of animals. The analysis of histological sections and comparison of the groups treated with the extracts and negative control presented no statistical difference, showing that TF and TC do not have an inductive effect on fibroblasts in the deposition of extracellular matrix.

On the other hand, the release of ROS by neutrophils during the inflammatory phase plays a fundamental role in the defense against microorganisms. However, excessive ROS can impede protease inhibitors that normally protect cells and extracellular matrix. Therefore, the use of agents that act as antioxidants can aid the healing process, by reducing the harm caused by ROS [35]. Comparing the data obtained from DPPH assay with cutaneous wound healing in rats, one may note that, despite the power of TF and TC scavenge free radicals, there was no enhancement of collagen deposition. This suggests that antioxidant activity of* L. thymoides* did not influence the healing of wounds or was not enough, at the concentration tested, to neutralize the excess of ROS formed by infiltrating neutrophils. Another aspect that might improve healing is the reduction or elimination of microorganisms in the wound. The TF and TC extracts showed no significant antimicrobial activity, but their respective dichloromethane fractions (TFD and TCD) and ethyl acetate (TFA and TCA) were effective against pathogens that commonly cause skin infections. Thus, it can be inferred that the use of* L. thymoides* in folk medicine for the treatment of wounds may be related to antiseptic effect on microorganisms at the site of the injury but not to the stimulation of healing by induction of fibroblasts and collagen deposition.

*L. thymoides* is also indicated in folk medicine to treat fever. Comparing this information with data obtained in this study, it was observed that TF and TC reduced, in dose-dependent manner, the rectal temperature of young rats with hyperthermia induced by yeast, with TF being more effective than TC. At a dose of 1200 mg/kg TF reduced rectal temperature of animals similar to that observed by the administration of dipyrone at 240 mg/kg. Although the dose of TF that required to reduce the temperature of animals with fever was five times higher than that of dipyrone, it should be considered that the crude plant extracts are a mix of incalculable secondary metabolites and varied concentrations. As TF effect was dose-dependent, it can be considered that the active principle responsible for* L. thymoides* antipyretic activity is much lower concentration in TF and TC, necessitating a high dose to observe the effect. Moreover, the oral administration was chosen because the use of this plant is via the preparation of tea for the treatment of fever. The oral administration of the extract may have caused the reduction of plasma levels of active principle needed for fever reduction, since the first pass effect that occurs in the liver metabolism may have metabolized a significant fraction of the substances in the extracts. The antipyretic action of substances occurs due to both peripheral and central effects. Thus, the reduction of the production of pyrogenic cytokines such as interleukin-1*β* and tumor necrosis factor alpha (TNF-*α*) and the interference on the peripherally leukocyte adhesion and reduced levels of prostaglandin E_2_ (PGE_2_) for inhibition of cyclooxygenase-2 (COX-2) in the preoptic area of the anterior hypothalamus (POHA) are considered the main targets of action of antipyretic drugs available in therapy [36, 37]. With this consideration, the crude extract from leaves of* L. thymoides* can act on two levels: (1) reducing the production of inflammatory mediators from peripheral that act as endogenous pyrogens and/or (2) reducing the production of PGE_2_ in POHA by inhibiting COX-2. In the latter case, there is the need of the active principle across the blood-brain barrier, which justifies the higher dose of TF to reach the appropriate concentration at the site of action. Thus, the data obtained with the animal model of hyperpyrexia-induced fever validates the use of* L. thymoides* in folk medicine to treat fever, excluding the possible toxic effects since TF at dose of 5 g/kg was not lethal for animals, nor was observed any change in the behavior of mice.

## 5. Conclusions

The results presented in this work show that the crude extracts and fractions from leaves and stems from* Lippia thymoides* have high contents of phenolic substances, presenting radical-scavenging and antimicrobial activity against Gram-positive bacteria, the dichloromethane, and ethyl acetate fractions being more potent than the crude extracts. The absence of healing stimulation of TF and TC indicates that popular use of* Lippia thymoides* is related to the antiseptic effect. In baker yeast-induced fever in young rats, TF reduced rectal temperature in a dipyrone-like effect, indicating that the active substance is more concentrated in the leaves of* Lippia thymoides*. Although the dose of TF has been too high, the lack of observable toxicity at a dose of 5 g/kg indicates that the reduction in rectal temperature was not due to toxicity. In conclusion, this work validates the popular use of* Lippia thymoides* for the treatment of wounds and fever, providing a source for biologically active substances.

## Figures and Tables

**Table 1:** Codes and yields of fractions obtained by liquid-liquid partition from crude extract of leaves (TF) and stems (TC) of *L*. *thymoides*.

Crude extract	Solvent	Fraction code	Yield (%)
TF	Hexane	TFH	5.4
	Dichloromethane	TFD	8.7
	Ethyl acetate	TFA	3.6

TC	Hexane	TCH	3.0
	Dichloromethane	TCD	13.7
	Ethyl acetate	TCA	3.9

**Table 2:** Content of total phenolics and antioxidant activity of crude extracts and fractions from leaves and stems of *L*. *thymoides*. Results expressed as mean ± SEM of three independent analyses.

Sample	Total phenolic content (mg/g GAE)	DPPH scavenging (EC_50_, *μ*g/mL)	*β*-carotene bleaching (%)
TF	115.5 ± 1.7	15.4 ± 1.6	9.3 ± 2.6
TFH	15.5 ± 0.7	333.7 ± 92.6	31.7 ± 3.2
TFD	90.5 ± 1.0	22.1 ± 2.5	29.3 ± 6.0
TFA	182.8 ± 2.4	5.7 ± 0.4	37.9 ± 4.1
TC	85.5 ± 4.4	18.4 ± 2.4	3.7 ± 2.5
TCH	14.5 ± 2.0	585.1 ± 186.6	9.4 ± 1.0
TCD	84.2 ± 6.1	14.5 ± 1.4	12.8 ± 0.4
TCA	213.6 ± 6.1	4.5 ± 0.2	0
AcA	—	5.1 ± 1.1	17.8 ± 5.6
BHT	—	13.3 ± 1.0	NT
BHA	—	4.0 ± 0.6	NT
QUE	—	NT	36.2 ± 11.6
PIR	—	NT	4.9 ± 1.7

mg/g GAE, mg gallic acid equivalents per gram; EC_50_, concentration that caused 50% of the DPPH radical scavenging; NT, nontested; TF, crude methanolic extract from leaves; TFH, hexane fraction from TF; TFD, dichloromethane fraction from TF; TFA, ethyl acetate fraction from TF; TC, crude methanolic extract from stems; TCH, hexane fraction from TC; TCD, dichloromethane fraction from TC; TCA, ethyl acetate fraction from TC; AAc, ascorbic acid; BHT, butylated hydroxytoluene; BHA, butylated hydroxyanisole; QUE, quercetin; PYR, pyrogallol.

**Table 3:** Antimicrobial activity of the crude extracts and fractions from leaves and stems of *L*. *thymoides*.

Sample	Microorganisms (MIC e MMC em mg/mL)															
	*E. coli *		*S. aureus *		*S. aureus *		*S. choleraesuis *		*B. cereus *		*M. luteus *		*C. albicans *		*C. parapsilosis *	
	CCMB 261		CCMB 262		CCMB 263		CCMB 281		CCMB 282		CCMB 283		CCMB 286		CCMB 288	
	MIC	MMC	MIC	MMC	MIC	MMC	MIC	MMC	MIC	MMC	MIC	MMC	MIC	MMC	MIC	MMC
TF	5.04	5.04	5.04	5.04	5.04	5.04	5.04	9.94	4.97	4.97	5.04	5.04	9.94	9.94	4.97	4.97
TFH	5.55	<11.1	1.39	2.77	2.77	2.77	5.55	11.1	2.77	2.77	5.55	<11.1	9.94	9.94	2.48	4.97
TFD	2.77	5.55	0.35	0.69	0.17	1.39	2.77	5.55	0.17	0.17	0.69	<11.1	2.48	4.97	0.62	1.28
TFA	2.77	2.77	0.35	0.69	0.35	0.69	1.39	1.39	0.69	0.69	0.69	<11.1	4.47	9.94	4.97	9.94
TC	2.56	5.12	0.62	2.56	2.56	5.12	2.56	2.48	2.48	2.48	2.56	5.12	<9.94	<9.94	9.94	9.94
TCH	2.77	11.1	2.77	5.55	2.77	5.55	5.55	11.1	2.77	2.77	2.77	<11.1	9.94	9.94	2.48	4.97
TCD	2.77	5.55	0.35	0.69	0.69	1.39	2.77	2.77	0.69	0.69	0.69	1.39	9.94	9.94	4.97	9.94
TCA	5.00	10.00	0.31	1.25	0.62	1.25	1.28	2.48	0.62	1.28	1.25	1.25	9.94	4.97	4.97	4.97
CLO	0.675	—	0.003	—	0.084	—	0.005	—	0.005	—	0.003	—	NT	—	NT	—
NIS	NT		NT		NT		NT		NT		NT		1.25	—	2.50	—
DMSO	5.55	—	5.55	—	11.1	—	5.55	—	5.55	—	5.55	—	NT	—	NT	—

MIC, minimum inhibitory concentration; MMC, minimum microbicidal concentration; NT, nontested; TF, crude methanolic extract from leaves; TFH, hexane fraction from TF; TFD, dichloromethane fraction from TF; TFA, ethyl acetate fraction from TF; TC, crude methanolic extract from stems; TCH, hexane fraction from TC; TCD, dichloromethane fraction from TC; TCA, ethyl acetate fraction from TC; CLO, chloramphenicol; NIS, nystatin.
